# Natural and evolved membrane-associated accessory proteins differentially engage SNARE machinery for AAV egress

**DOI:** 10.1128/jvi.00026-26

**Published:** 2026-06-10

**Authors:** Robert M. Fusco, Joshua A. Hull, Chenxuan Tong, Xinlong Wan, Zachary C. Elmore, Aravind Asokan

**Affiliations:** 1 Department of Biomedical Engineering, Duke University 3065, Durham, North Carolina, USA; 2 Department of Surgery, Duke University School of Medicine 12277 , Durham, North Carolina, USA; 3 Department of Molecular Genetics and Microbiology, Duke University School of Medicine 12277 , Durham, North Carolina, USA; Cornell University Baker Institute for Animal Health, Ithaca, New York, USA

**Keywords:** Golgi transport, secretory pathway, SNARE machinery, viral egress, parvovirus, adeno-associated virus

## Abstract

Adeno-associated viruses (AAV) are non-enveloped parvoviruses widely utilized for therapeutic gene delivery. The membrane-associated accessory protein (MAAP), a key viral protein, is critical for cellular egress of both infectious and recombinant AAV particles. However, the structure-function correlates of this process have remained largely elusive. Here, we applied structure-guided evolution to enhance AAV egress and dissect the biology of both natural and synthetic MAAP (synMAAP) variants. Three structurally distinct combinatorial libraries focused on disordered domains within MAAP8 (derived from AAV serotype 8) were subjected to selection through both transfection and infectious cycling. Newly enriched synMAAPs obtained as a function of enhanced AAV secretion from host cells revealed a profound bias toward mutations within the C-terminal domain. The most potent synMAAPs showed increased propensity to form extended alpha-helix structures as predicted by structural modeling, amphipathicity, and hydrophobicity scores. Confocal microscopy demonstrated that, unlike natural MAAP8, synMAAPs are excluded from the nucleus and exhibit strong perinuclear accumulation within the Golgi. Proximity ligation analysis, biochemical analysis, and gene deletion studies further corroborated that synMAAPs appear to more efficiently engage SNARE complex proteins, notably involving VAMP3, to potentiate AAV egress. Together, these findings reveal new insights into structure-function correlates of viral egress from host cells and may help improve recombinant vector production for gene therapy applications.

**IMPORTANCE:**

Viruses must exit infected cells to spread, yet this process is poorly understood for adeno-associated virus. In this study, we examine how adeno-associated virus exits mammalian cells without causing cell death. By engineering and evolving new versions of the viral egress factor, we identify cellular trafficking pathways that are exploited during viral release. These findings improve our understanding of how viruses interact with host cells. Additionally, the engineered egress factors described in this study may be used to improve the efficiency of adeno-associated virus production for gene therapy applications.

## INTRODUCTION

Cellular egress is a critical component of the viral lifecycle, enabling the dissemination of progeny virions. Many viruses exit the cell through lytic mechanisms such as apoptosis or necrosis, leading to the release of intracellular contents. Alternatively, some viruses exploit a range of host secretory pathways to achieve non-lytic egress, including conventional exocytosis, lysosomal exocytosis, secretory autophagy, exosome secretion, or direct budding from the plasma membrane (1–5). These mechanisms, while sometimes stochastic, often rely on specific viral proteins that orchestrate egress, regardless of cytopathic effect. For example, the norovirus NS3 protein has been reported to induce programmed cell death by mimicking host MLKL function and targeting the mitochondria (6). Poliovirus, traditionally considered a lytic virus, can also undergo non-lytic release via a vesicle-mediated pathway in which the nonstructural protein 3CD has been implicated (7). In hepatitis E virus, the pORF3 protein tethers to the surface of MVBs through TSG101 binding, facilitating the incorporation of virions and promoting their release (8). These examples emphasize the diverse roles that viral proteins play in modulating viral egress. Because progeny release is a core dependency of the viral life cycle, these molecules also represent attractive targets for antiviral intervention. A deeper understanding of how viral and host factors interact to coordinate egress may enable the rational design of antivirals tailored to specific pathogens.

A particularly interesting example in this regard is adeno-associated virus (AAV)—a small, non-enveloped member of the *Parvoviridae* family, genus *Dependovirus,* which depends on co-infection by a helper virus such as Adenovirus (Ad) or Herpes Simplex Virus (HSV) for propagation (9). The ~4.7 kb single-stranded DNA genome packaged within the icosahedral T = 1 capsid contains four overlapping genes (*rep, cap, aap,* and *maap*) that encode nine distinct proteins through alternative splicing and alternative open reading frames (9, 10).

The membrane-associated accessory protein (MAAP), encoded by the *maap* ORF, is known to play a critical, yet incompletely understood role in AAV biology and recombinant vector production (10–13). The MAAP encoding ORF is contained within the *cap* region of common AAV serotypes 1-12, rh.8, rh.10, rh32.33, along with bovine and porcine AAVs (11, 14). While this protein lacks homology to any known eukaryotic, prokaryotic, or viral proteins, from a structural perspective, MAAP contains a disordered N-terminal domain followed by a hydrophobic region and a C-terminal amphipathic domain linked through a T/S-rich region (11, 12, 14). Functionally, MAAP appears to broadly impact AAV particle replication and egress in infected host mammalian cells. Furthermore, MAAP also appears to promote the association of secreted AAV particles with extracellular vesicles (EVs) (11). However, mechanistic insights into MAAP-mediated egress have remained largely elusive.

In the current study, we utilized a structure-guided evolution approach to engineer synthetic variants of MAAP8, derived from a non-human primate AAV isolate (AAV serotype 8), previously shown to be robustly secreted from mammalian cells (15, 16). To dissect specific structural determinants, we targeted three distinct secondary structural domains within the MAAP N- and C-terminal regions as well as the linker domain for saturation mutagenesis. We then subjected these distinct MAAP libraries to evolutionary pressure through infectious cycling of secreted AAV particles and selected new, synthetic MAAP (synMAAP) variants that potentiate AAV egress. Next, we sought to define how synMAAPs localize within the cell and interact with cellular trafficking machinery in comparison with natural parental MAAP. Our battery of approaches, including structural modeling, confocal imaging, and proteomic analyses, yielded new mechanistic insights into MAAP interactions with specific members of the SNARE complex, thereby shedding light on viral egress pathways in general. Moreover, these potent synMAAP molecules may have applications in recombinant AAV manufacturing technologies.

## MATERIALS AND METHODS

### Structure-guided generation of MAAP libraries

Combinatorial variant libraries were synthesized by Twist Biosciences and cloned into an ITR plasmid containing a multiple cloning site, an hEF1α core promoter, and a bGH poly(A) signal, with a stuffer sequence to obtain a 4.3 kb ITR-to-ITR length. Libraries were designed with AgeI and BamHI restriction sites, and both the plasmid and libraries were digested with AgeI and BamHI. Following gel electrophoresis (1.2% agarose) and DNA recovery, fragments were ligated using T4 DNA Ligase (New England Biolabs) at a 1:3 vector to insert molar ratio. Reactions were incubated overnight at 16°C prior to transformation. Notably, the *maap* ORF start codon was mutated from the endogenous CTG to the canonical ATG. Variant amino acids for each position along mutated regions represented 95% of the sequence pool, and the wild type represented the remaining 5% to allow for tracking of variant enrichment over subsequent passages.

Theoretical library diversity was 5.12E11 variants per library. Following cloning and transformation into electrocompetent Max Efficiency DH10β (ThermoFisher Scientific), plasmid DNA was prepared using a MaxiPrep kit (Zymo Research). Next-generation sequencing (NGS) was used to evaluate library diversity and confirmed 6.8E4 unique variants for library 1, 4.8E4 unique variants for library 2, and 7.3E4 unique variants for library 3.

### Recombinant AAV vector production

Recombinant AAV vectors were produced as previously described (17). For suspension platform production, HEK293G cells (generated in-house at Duke University) were grown to a density of 2E6 cells/mL in 250 mL shake flasks containing 100 mL of freshly prepared F-17 medium containing 2% GlutaMAX (wt/vol), 1% pen-strep (vol/vol), and 0.1% poloxamer 188 (vol/vol). Then, cells were transfected with plasmids encoding Ad helper genes (pHelper; 0.6 μg/mL), rep/cap genes (pRepCap; 0.5 μg/mL), and the necessary transgene (pTR; 0.3 μg/mL) using polyethylenimine (PEI; PolyScience) at a ratio of 3:1 (PEI to DNA). Supernatant and cells were harvested 6 days post-transfection unless otherwise noted.

For adherent cell production, HEK293 cells were cultured in Dulbecco’s Modified Eagle’s Medium (DMEM) supplemented with 10% heat-inactivated fetal bovine serum (FBS), 100 U/mL penicillin, and 100 μg/mL streptomycin. Cells were incubated at 37˚C at 5% CO_2_. Recombinant AAV vectors were produced using triple transfection as previously described. Briefly, HEK293 cells were seeded to be 70%–80% confluent at the time of transfection. Using PEI at a ratio of 3:1 PEI:DNA ratio, cells were transfected with plasmids encoding Ad helper genes (pHelper), rep/cap genes (pRepCap), and the necessary transgene (pTR) at a ratio of 1.2:1:0.6 (wt/wt/wt). Similarly, for transcomplementation of MAAP, cells were transfected with pHelper, pRepCapΔMAAP, pMAAP, and pTR at a ratio of 1.2:1:1:0.6 (wt/wt/wt/wt). Supernatant was harvested at days 4 and 6, unless otherwise noted. Cells were harvested on day 6.

### Downstream processing of AAV vectors

For subsequent processing and purification of AAV vectors, media supernatant was precipitated by incubating with 12% polyethylene glycol 8000 (PEG; Millipore-Sigma) overnight at 4°C. Cell pellets were lysed by three freeze-thaw cycles. PEG pellets and clarified cell lysate were treated with 20 μL DNaseI (10 mg/mL; multiple vendors) before purification by iodixanol (Millipore-Sigma) ultracentrifugation.

For iodixanol purification, gradients were prepared by layering 3 mL 17% iodixanol, 3 mL 25% iodixanol, 4 mL 40% iodixanol, and 3 mL 60% iodixanol in clear 17 mL ultracentrifuge tubes (Beckman-Coulter). Resuspended PEG pellets, lysate, or a mixture of both were overlaid onto the gradient and centrifuged at 30,000 rpm for >15 hours. The first two layers were discarded, and 550 μL fractions were collected.

For affinity purification, supernatants were clarified by centrifugation (1,000 × *g* for 10 minutes) and transferred to new tubes. POROS CaptureSelect AAVX resin (“AAVX,” ThermoFisher Scientific; binding capacity >1E13 vg/mL of resin) was pelleted (2,500 × *g* for 10 minutes), washed three times with PBS, resuspended in 1 mL of PBS, and added to the clarified supernatant. Samples were incubated for 1 hour, rocking at room temperature. The slurry was transferred to Econo-Pac Chromatography Columns (Bio-Rad) and allowed to drain by gravity. Columns were washed with five column volumes of PBS, and the bound vector was eluted with 3 column volumes of 0.1M glycine (pH 3.0) into tubes preloaded with 0.1 volume of neutralization buffer (1M Tris-HCl, pH 8.5). Eluate was immediately buffer exchanged using Pierce Protein Concentrators (100K MWCO; ThermoFisher Scientific). Samples were diluted to 50 mL in formulation buffer (PBS supplemented with 1 mM MgCl_2_ and 0.001% poloxamer 188 non-ionic surfactant), concentrated to 1 mL by centrifuging at 2,500 × *g,* re-diluted to 20 mL, and concentrated to 1 mL.

Iodixanol fractions or buffer-exchanged AAVX eluates were titered as described below. Peak fractions were pooled, and buffer exchanged using the same protocol. Final genome concentration was determined by qPCR and stored at 4°C short term or −80°C long term.

### Quantification of AAV genomes

AAV vector genome titers were quantified using quantitative PCR (qPCR) with primers targeting the inverted terminal repeats (ITRs). Viral preparations, including purified virus, conditioned supernatant, or clarified cell lysate, were first treated with DNase I (0.1 mg/mL final concentration) and incubated at 37°C for 1 hour to remove unencapsidated genomes. Following DNase treatment, samples were treated with 6 μL of 0.5M EDTA to inactivate the DNase, diluted 1:2 in 10% Tween-20 (vol/vol), then 1:100 in nuclease-free water before being analyzed using a ThermoFisher QuantStudio 3 real-time PCR system. Vector genome copy numbers were determined by interpolation from a standard curve using AAV samples of known titer.

### Structure-guided selection and evolution of MAAP libraries

To capture a broad range of variants while also applying selective pressure, two parallel evolution tracks were used. The transfection-based track enriches variants based on their expression and resulting secretion, without any packaging or infectivity constraints. Conversely, the serial viral passaging track applies greater stringency by sampling the same variant pool over time and enriching for variants capable of completing the entire viral lifecycle.

#### Transfection-mediated selection

Purified parental virus packaging the individual MAAP libraries were produced in suspension cells as described above, with the exception that only supernatant was collected at day 3 to apply selection stringency. During the iodixanol fractionation step, 2 μL of the pooled peak fractions of iodixanol was placed into a Q5 PCR in place of DNA and amplified using standard PCR according to the manufacturer’s instructions. The subsequent PCR was separated using a 1% agarose gel. Banding corresponding to the appropriate size was excised, and the DNA was recovered using the Gel DNA Recovery Kit (Zymo Research). This amplified library was then cloned into the original ITR-containing backbone using HiFi Assembly (NEB), and the plasmid was prepared using the methods described in “Library Generation” above. Next-generation sequencing, described below, was then used for determining variant enrichment.

#### Infectious cycling by serial viral passaging

Purified parental virus packaging the individual MAAP libraries (above) was added directly to suspension cells at a multiplicity of infection (MOI) of ≤1,000. After 24 hours of transduction, the helper plasmid and pRepCapΔMAAP were transfected into the culture to induce viral replication and packaging. After 24 hours of transfection, the media was changed to remove the input virus. An additional 24 hours post-media change, the new media was collected for NGS analysis, as described below.

### Next-generation sequencing of enriched variants

For NGS, viral preparations were treated with DNase I, as described above, to remove unencapsidated genomes (parental plasmid was not DNase I treated). The vector pool was amplified by PCR and sequenced using Azenta Amplicon EZ service as previously described. The sequences were then collated, and fold-change was determined based on the sequence abundance compared to previous rounds of evolution.

### AAV capsid quantification by ELISA

Total AAV capsid concentrations were determined by ELISA. Crude supernatant was loaded onto a Progen AAV9 ELISA plate coated with anti-capsid antibodies in accordance with the manufacturer’s instructions. After incubation and washing, detection was performed using an HRP-conjugated secondary antibody and a colorimetric substrate. Absorbance was measured at 450 nm using the VarioSkan LUX microplate reader (ThermoFisher Scientific). Capsid concentration was calculated based on a standard curve provided by the manufacturer.

### Mass photometry

Mass photometry was performed using a Refeyn SamuxMP to assess the particle heterogeneity of AAV preparations. Standards and samples were diluted to an appropriate concentration in formulation buffer and applied to a clean microscope coverslip. Molecular weights were measured and analyzed using the Refeyn DiscoverMP software.

### Proximity ligation assay

HEK293 cells were transfected with pRepCap8ΔMAAP, CMV-MAAP8-13×BioID2-HA, pHelper, and pTR using PEI at a ratio of 3.5 μL per μg of DNA. Forty-eight hours post-transfection, the supernatant was supplemented with 50 mM biotin to allow for proximity-related biotinylation of interacting proteins. Cells were harvested 72 hours post-transfection for the subsequent analyses. Cells were lysed in a urea buffer containing 8M urea, 50 mM Tris-HCl (pH 7.5), 1 mM DTT, and 1× HALT protease inhibitor cocktail. Lysates were supplemented with 0.2 volumes of 20% Triton X-100 and sonicated. Following clarification, biotinylated proteins were purified using High-Capacity Streptavidin Agarose. Beads were washed in urea buffer, and proteins were eluted in 25 mM Tris-HCl, 50 mM NaCl, 10 mM DTT, 2% SDS, and 3M biotin.

### Mass spectrometry sample preparation and analysis

Samples were spiked with undigested bovine casein at a total of either 120 or 240 fmol as an internal quality control standard. Next, samples were supplemented with 20% SDS, reduced with 10 mM dithiolthreitol for 30 minutes at 80°C, alkylated with 20 mM iodoacetamide for 30 minutes at room temperature, then supplemented with a final concentration of 1.2% phosphoric acid and 720 μL of S-Trap (Protifi) binding buffer (90% MeOH/100 mM TEAB). Proteins were trapped on the S-Trap microcartridge, digested using 20 ng/μL sequencing grade trypsin (Promega) for 1 hour at 47°C, and eluted using 50 mM TEAB, followed by 0.2% FA, and lastly using 50% ACN/0.2% FA. All samples were then lyophilized to dryness. Samples were resolubilized using 120 μL of 1% TFA/2% ACN with 25 fmol/μL yeast ADH.

Quantitative LC/MS/MS was performed on 2 μL using a nanoAcquity UPLC system (Waters Corp) coupled to a Thermo Orbitrap Fusion Lumos high-resolution accurate mass tandem mass spectrometer (ThermoFisher Scientific) equipped with a FAIMSPro device via a nanoelectrospray ionization source. Briefly, the sample was first trapped on a Symmetry C18 20 mm × 180 µm trapping column (5 μL/min at 99.9/0.1 vol/vol water/acetonitrile), after which the analytical separation was performed using a 1.8 μm Acquity HSS T3 C18 75 μm × 250 mm column (Waters Corp.) with a 90-minute linear gradient of 5%–30% acetonitrile with 0.1% formic acid at a flow rate of 400 nanoliters/minute (nL/min) with a column temperature of 55°C. Data collection on the Fusion Lumos mass spectrometer was performed for three different compensation voltages (−40 V, −60 V, and −80 V). Within each CV, a data-dependent acquisition (DDA) mode of acquisition with a *r* = 120,000 (@ *m*/*z* 200) full MS scan from *m*/*z* 375 to 1,500 with a target AGC value of 4e5 ions was performed. MS/MS scans were acquired in the ion trap in Rapid mode with a target AGC value of 1e4 and max fill time of 35 ms. The total cycle time for each CV was 0.66 seconds, with total cycle times of 2 seconds between like full MS scans. A 20-second dynamic exclusion was employed to increase the depth of coverage. The total analysis cycle time for each injection was approximately 2 hours.

#### Quantitative mass spectrometry data analysis

Following UPLC-MS/MS analyses, data were imported into Proteome Discoverer 2.5 (ThermoFisher Scientific), and individual LCMS data files were aligned based on the accurate mass and retention time of detected precursor ions (“features”) using the Minora Feature Detector algorithm in Proteome Discoverer. Relative peptide abundance was measured based on peak intensities of selected ion chromatograms of the aligned features across all runs. The MS/MS data were searched against the SwissProt *H. sapiens* database (downloaded in November 2019), a common contaminant/spiked protein database (bovine albumin, bovine casein, yeast ADH, etc.), and an equal number of reversed-sequence “decoys” for false discovery rate determination. Sequest with Infernys enabled (v 2.5, Thermo PD) was utilized to produce fragment ion spectra and to perform the database searches. Database search parameters included fixed modification on Cys (carbamidomethyl) and a variable modification on Met (oxidation). Precursor mass tolerances were 2.0 ppm, and product ion mass tolerances were 0.8 Da, with full trypsin enzyme rules required. Peptide Validator and Protein FDR Validator nodes in Proteome Discoverer were used to annotate the data at a maximum 1% protein false discovery rate based on *q*-value calculations. Note that peptide homology was addressed using razor rules in which a peptide matched to multiple different proteins was exclusively assigned to the protein that had more identified peptides. Protein homology was addressed by grouping proteins that had the same set of peptides to account for their identification. A master protein within a group was assigned based on % coverage.

Prior to imputation, a filter was applied such that a peptide was removed if it was not measured in at least two unique samples (50% of a single group). After that filter, any missing data values were imputed using the following rules: (i) if only a single signal was missing within the group of three, an average of the other two values was used or (ii) if two out of three signals were missing within the group of three, a randomized intensity within the bottom 2% of the detectable signals was used. A normalization was then applied to the data by excluding the highest and lowest 10% of the signals and then making the average of the remaining signals be the same across all samples. To summarize to the protein level, all peptides belonging to the same protein were summed into a single intensity.

#### STRINGdb analysis parameters

The Duke Proteomics and Metabolomics Core set the enrichment cutoff at a fold-change of 1.5. Proteins with a fold change exceeding this threshold were analyzed in STRINGdb (string-db.org) using the following settings: Network type: physical subnetwork; meaning of network edges: evidence; minimum required interaction score: high confidence (0.700); network display options: hide disconnected nodes in the network. All other parameters remained unchanged.

### Co-immunoprecipitation

Cells were seeded in 15 cm plates at 1.5E5 cells/mL. Three days later, cells were transfected with 5 μg of MAAP and 5 μg of pGFP-VAMP3 using PEI at a ratio of 3:1. Immunoprecipitation was performed as previously published with some minor alterations (18). Two days post-transfection, cells were harvested and lysed in lysis buffer (50 mM Tris-HCl, pH 8.0, 150 mM NaCl, 1% NP-40, and 1× HALT protease inhibitor). Lysate was clarified by centrifugation at max speed for 10 minutes at 4°C. The supernatant was collected and incubated with anti-HA beads for 60 minutes at room temperature. Supernatant was removed, and beads were washed four times in wash buffer (50 mM Tris-HCl, 150 mM NaCl, 0.5% Tween-20). Samples were eluted in 0.15M glycine, pH 3.0, for 10 minutes before being neutralized with 1M Tris, pH 8.0. Samples were analyzed by Western blot for immunoprecipitation.

### SDS-PAGE and western blot analysis

Samples were mixed with LDS supplemented with 2.5% β-mercaptoethanol and heated at 95°C for 5 minutes prior to electrophoresis. Proteins were separated by SDS-PAGE using 4%–12% polyacrylamide gels (BioRad) in 1× Tris/Glycine/SDS buffer at 140 V for 45 minutes. Gels were processed for Western blotting and transferred onto PVDF membranes using a semi-dry transfer system. For Western blotting, membranes were blocked in a 5% milk solution for 1 hour at room temperature, then incubated with primary antibodies against HA (1:10,000) or GFP (1:1,000) overnight at 4˚C. After washing in TBST, membranes were incubated with HRP-conjugated secondary antibody (α-mouse 1:10,000), washed again, and developed using the SuperSignal West Femto Maximum Sensitivity Substrate (ThermoFisher Scientific).

### Generation of lentiviral gene knockout cell lines

HEK293 cells were seeded in six-well assay plates at a density of 5e5 cells/mL and grown at 37°C, 5% CO_2_ for 24 hours. Cells were transfected with lentiCRISPRv2 using PEI, with three guides per gene (500 ng per plasmid). After puromycin selection, cells were passaged to allow for protein turnover before use in secretion assays.

### Immunofluorescence and confocal microscopy

HEK293 cells were seeded at 2e5 cells/mL in an 18-well ibiTreat chamber coverslip (ibidi). Cells were transfected using Lipofectamine 3000 (ThermoFisher Scientific) with a total of 100 ng of plasmid per the manufacturer’s protocol. For immunofluorescence, cells were fixed with 4% paraformaldehyde (multiple vendors) in PBS for 15 minutes at room temperature. Following fixation, cells were permeabilized with 0.5% Tween-20 for 5 minutes. Cells were blocked in 1% bovine serum albumin in PBS supplemented with 0.5% Tween-20 for 30 minutes. Then, cells were incubated with the primary antibody overnight at 4°C. After three 5-minute washes, samples were incubated in the secondary antibody for 1 hour in the dark at room temperature. After another round of washes, nuclei were counterstained with DAPI (0.1 μg/mL) for 5 minutes.

For live cell imaging, 24 hours following transfection, BODIPY TR Ceramide (ThermoFisher Scientific) was resuspended according to the manufacturer’s instructions, then diluted 1:100 in serum-free cell culture media. Staining solution was added, and cells were incubated for 18 hours at 37°C. The morning of imaging, nuclei were stained using Hoechst 33342. Briefly, Hoechst was diluted to 1 μg/mL in phenol red-free DMEM and added to cells for 10 minutes at 37°C. Cells were washed with DMEM before proceeding to imaging.

Imaging was performed using a Zeiss 880 inverted confocal microscope. Z-stack and single-plane images were acquired. For image processing, channels were separated, and the Hoechst stain was used to generate a nuclear mask. The mask was then applied to the green channel to identify regions with a nuclear signal. The fluorescence intensity was quantified using corrected total cell fluorescence (CTCF).


CTCF=Integrated Density−(area of ROI ×mean background fluoresence)

For Pearson analyses of co-occurrence between Golgi and MAAP or VAMP and MAAP, images were masked on either the Golgi (red) or VAMP3 and analyzed using Just Another Colocalization Plugin (JACoP) in ImageJ.

## RESULTS

### Structure-guided evolution yields distinct classes of synthetic MAAP variants (synMAAPs) that potentiate AAV egress

**Fig 1:**
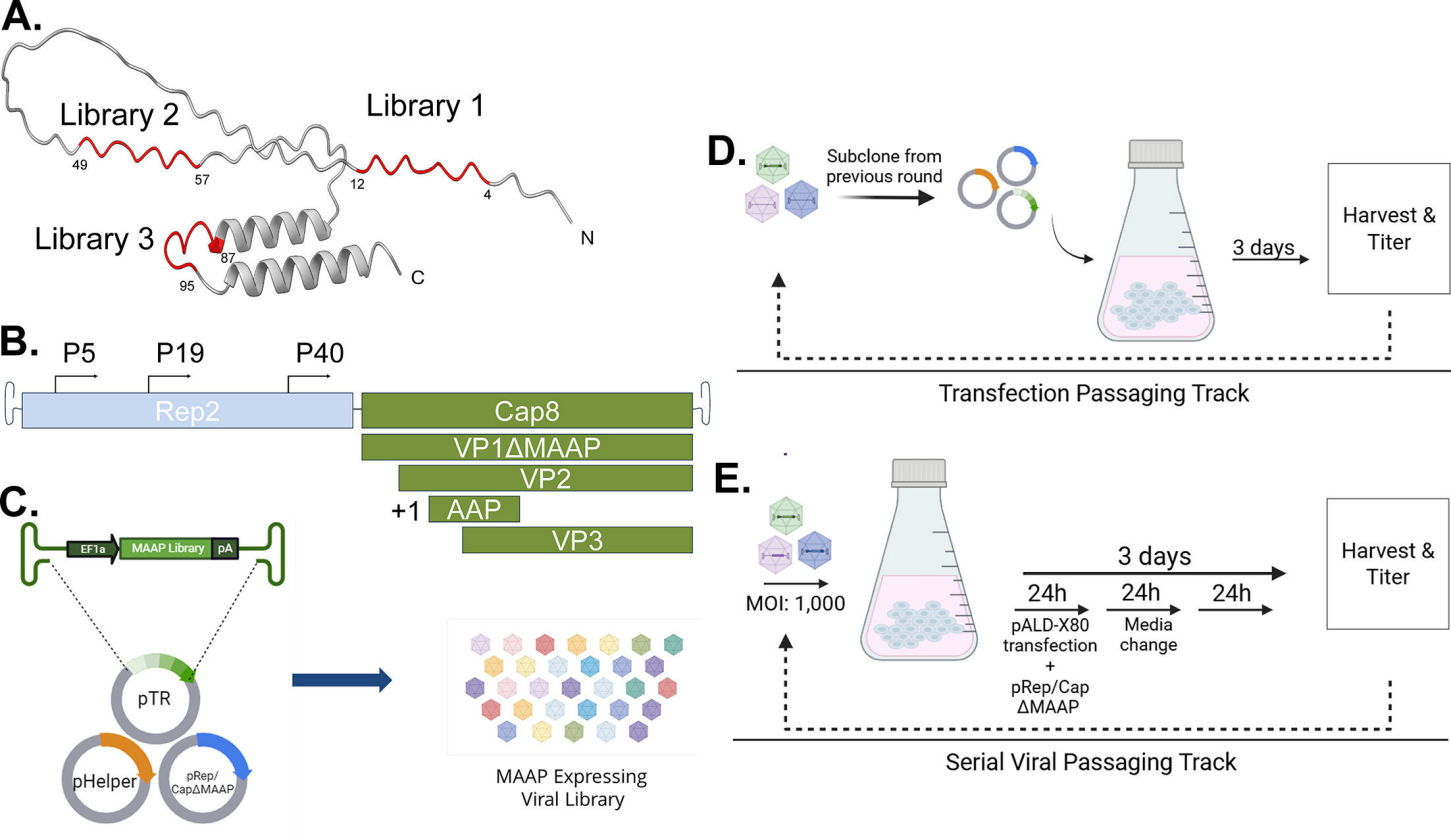
Library construction and experimental design of MAAP evolution. (**A**) AlphaFold3 structural prediction of the AAV8 MAAP, highlighting the three library regions chosen for mutagenesis (red). (**B**) Depiction of the AAV2/8 rep/cap plasmid used for directed evolution. MAAP was knocked out by mutating the start codon and introducing several silent stops in the MAAP +1 ORF (pRepCapΔMAAP). (**C**) Overview of the triple transfection plasmids. MAAP variants were cloned under transcriptional control of the ubiquitous human elongation factor 1α and co-transfected along with a helper plasmid and pRepCapΔMAAP. As such, each resulting virion contains a unique MAAP variant. (**D**) Graphical representation of cycling conditions for the transfection track, demonstrating subcloning of each viral pool post-production. (**E**) Graphical representation of the serial viral passaging (infectious cycling) track.

Structure-guided evolution has been widely used to engineer the AAV capsid to alter tropism and/or enable immune evasion (19–23). Here, using a structure-guided approach, we designed three saturation mutagenesis libraries of the AAV8 MAAP protein (MAAP8) (Fig. 1A). MAAP8 was chosen as the parental template for mutagenesis due to favorable attributes such as the ability to mediate early and efficient AAV egress as well as demonstrated trans-complementation of multiple serotypes in AAV production (11).

To evaluate functional variants, we chose three highly diverse regions of MAAP8 to modify: the N-terminus, the T/S-rich linker regions, and the region adjacent to the C-terminal α-helix. Sequence alignments of MAAPs across serotypes revealed diversity in the N-terminal region, providing the rationale for Library 1. In contrast, the T/S-rich linker and the region adjacent to the C-terminal α-helix were previously shown to be dispensable for viral secretion, motivating the diversification in Libraries 2 and 3 (Fig. 1A) (11). Given their diversity and dispensability in prior studies, we hypothesized that these regions might tolerate diversification that could reveal alternative mechanisms to support viral secretion.

To isolate the effect of the synthetic MAAP variants from naturally expressed MAAP, we generated a Rep/Cap plasmid with mutations that prevent endogenous MAAP expression while maintaining Cap function (Fig. 1B). Virus was produced in suspension by triple transfection and purified by iodixanol ultracentrifugation, creating a pool of individual AAV particles each encapsidating an individual MAAP variant (Fig. 1C). We used two complementary selection strategies to enrich for variants. First, we utilized a plasmid-based cycling system where MAAP sequences from secreted virions were amplified and re-cloned into ITR plasmids. Second, we utilized serial viral passaging, wherein secreted viral particles were directly applied to cells at an MOI of 1,000 and enriched through subsequent production cycles using helper gene supplementation (Fig. 1C through E). Through these complementary approaches, we identified thousands of enriched MAAP variants within each sub-family that mediated AAV secretion in a MAAP-null background. Having identified these pools, we next sought to determine which individual candidates exhibited improved AAV secretion efficiency.

### Newly evolved synMAAP variants display structural domain-dependent abilities to enable AAV egress

**Fig 2:**
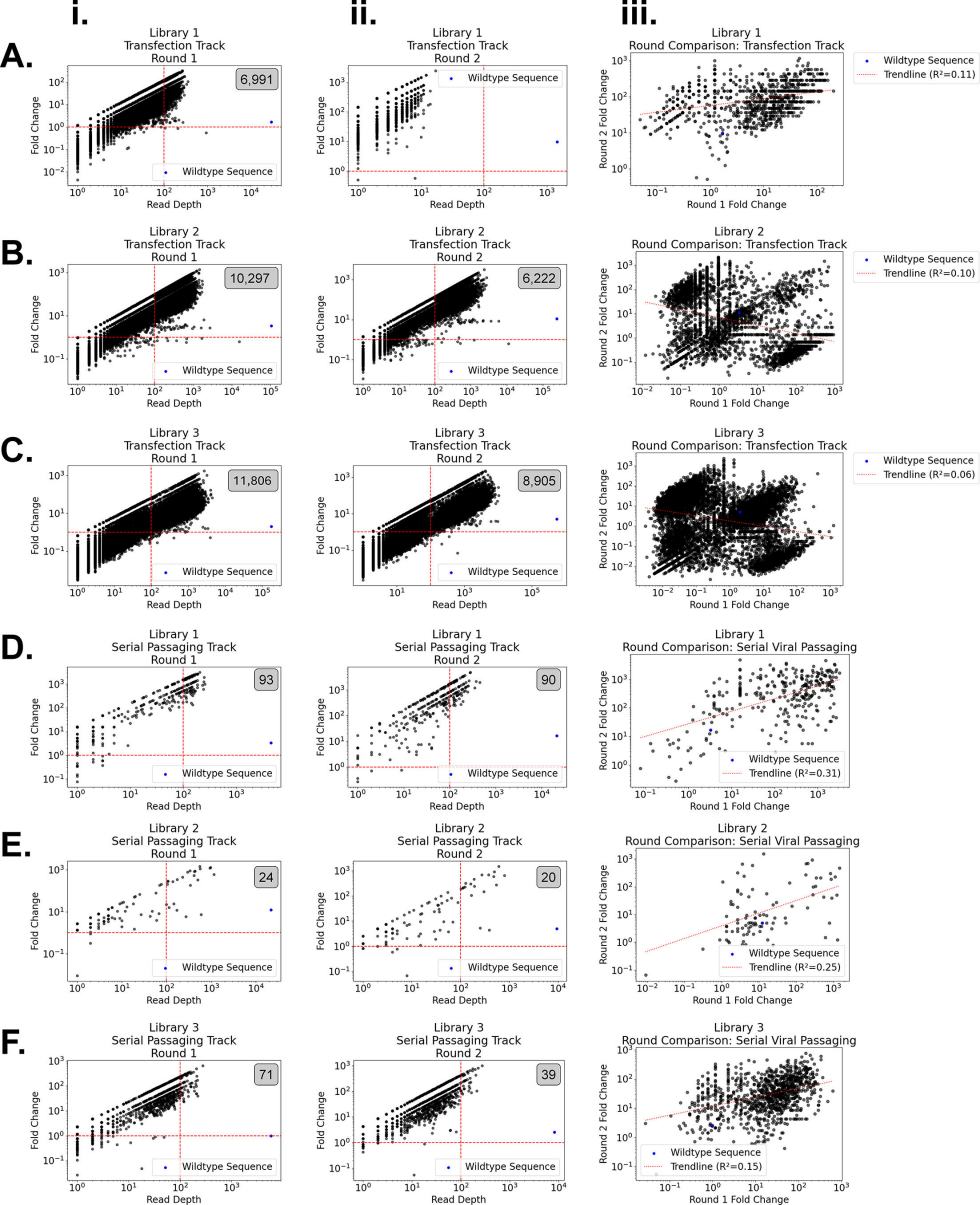
Transfection- and infection-based evolution strategies yield thousands of enriched engineered MAAP variants. Next-generation sequencing of MAAP libraries evolved by transfection-based cycling (**A–C**) and infectious cycling (**D–F**) for Library 1 (**A, D**), Library 2 (**B, E**), and Library 3 (**C, F**) across rounds 1 (i) and 2 (ii). Insets show the number of variants meeting enrichment thresholds (FC > 1 and read depth > 100). Panel (iii) compares rounds 1 and 2 for both cycling methods and *R*^2^.

The three MAAP8 libraries were designed to sample sequence diversity across distinct structural regions. NGS was used to track each round of library cycling, with candidate sequences evaluated based on fold-enrichment relative to the initial input and read depth. This approach enabled the identification of MAAP variants with the potential to enhance viral secretion. Most libraries from the transfection track generated a robust set of candidates displaying enhanced egress, with >6,000 individual candidates enriched for each cycle that displayed enrichment (Fig. 2Ai and Bi and ii, Ci and ii). To apply additional stringency and narrow the number of candidates to evaluate, we performed serial passaging of the secreted virus in parallel. We observed significantly fewer enriched variants in the viral passaging track compared to the transfection-based cycling (Fig. 2Di and ii, Ei and ii, Fi and ii). This is likely a result of the stronger selective pressure applied during repeated infection cycles. In turn, only variants that conferred robust secretion would arise. Analysis of multiple cycling rounds for both the transfection and serial passaging suggested they were largely uncorrelated (Fig. 2A–Fiii). While we evaluated candidates from all three libraries, candidates from Library 1, based on the disordered and highly divergent N-terminal domain only showed a modest increase in secretion. SynMAAP candidates selected from Library 2 based on the T/S-rich linker domain, previously shown to be dispensable, did not show a secretion advantage (Fig. S1). Slight differences in total titers between libraries can be attributed to different serotypes used during evaluation, with secretion by MAAP8 trans-complementation being generally consistent between serotypes and experiments.

**Fig 3:**
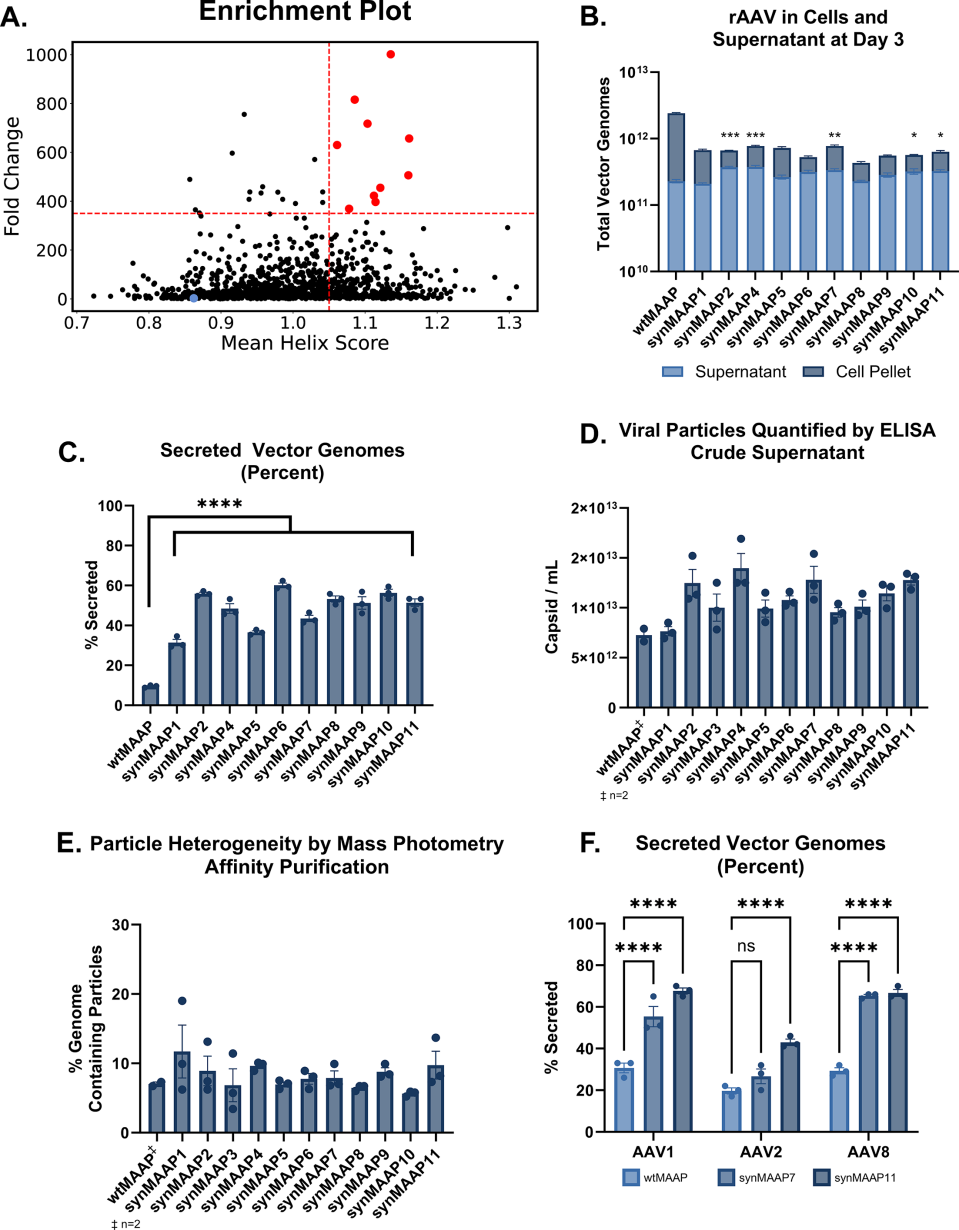
Evaluation of MAAP variants with predicted helix yield robust secretion profiles. (**A**) Enrichment of Library 3 variants after round 2 of passaging plotted against their Chou-Fasman mean helix scores. (**B**) Quantitative PCR titers of virus of secreted and cell-retained vector genomes at 3 days post-transfection. Select variants show increased secretion at 3 days post-transfection (statistics plotted), and all variants show a significant reduction in retained genomes (*P* ≤ 0.001). Significance was determined using two-way ANOVA, with Dunnett’s post-test. (**C**) Percentage of total vector genomes released into the supernatant. Significance was determined using one-way ANOVA, with Dunnett’s post-test. (**D**) Capsid abundance in crude supernatant was measured by AAV9 ELISA, demonstrating elevated capsid levels for several synMAAP variants. (**E**) Refyn mass photometric analysis showing % of full genome-containing AAV particles secreted into media supernatant following AAVX affinity purification. (**F**) Performance of top synMAAP variants during production of multiple AAV serotypes demonstrates a serotype-independent enhancement of secretion. Significance was determined using two-way ANOVA, with Dunnett’s post-test. Data are presented as mean values ± SEM. *P ≤ 0.05; **P ≤ 0.01; ***P ≤ 0.001; **** *P* ≤ 0.0001. *N* = 3 unless otherwise stated.

Given that the most robust increase in AAV secretion was seen for MAAP library-derived candidates based on the C-terminal domain (Library 3), we conducted further structural and biophysical analyses to determine the rationale for these observations. AlphaFold3 modeling predicted MAAP8 to be largely disordered except for the C-terminal region, leading us to rationalize that variants predicted to harbor predominantly α-helical domains may correlate with enhanced library selection (Fig. 1A). To prioritize candidates for individual evaluation, we applied the Chou-Fasman analysis to predict which variants were likely to extend the C-terminal helix, potentially improving folding, stability, or overall structure (24–26). According to this method, variants with a mean helix score greater than 1.03 are predicted to form an α-helix. To further refine the candidate pool, we applied cutoffs of ≥350-fold enrichment in the final cycling round and a mean helix score of ≥1.05 (Fig. 3A), resulting in 10 candidates for detailed functional evaluation. These variants were cloned into a mammalian expression cassette under control of a human elongation factor 1α promoter (EF1α) for further characterization. Protein expression in HEK293 cells was confirmed by Western blot (Fig. S2A).

### C-terminal domain-based synMAAP variants are robust potentiators of AAV egress

We first assessed vector titers at 3 days post-transfection to evaluate both secreted and cell-retained DNase-protected vector genomes (hereafter referred to as vector genomes, unless otherwise stated). All variants showed a significant decrease in retained vector genomes (0.1- to 0.2-fold lower), while several candidates (synMAAP2, synMAAP4, synMAAP6, synMAAP7, synMAAP10, and synMAAP11) exhibited a significant increase in secreted vector genomes (Fig. 3B). Calculating the percentage of secreted vector genomes revealed a 3- to 6.5-fold increase for all synMAAP candidates relative to wild-type MAAP trans-complementation (Fig. 3C).

We hypothesized that the reduction in intracellular titers results from rapid secretion of viral particles, including empty capsids, thereby limiting the availability of preformed particles for genome packaging. To test this hypothesis, we measured the total intact viral capsids from crude supernatant by AAV9 ELISA (Fig. 3D). Most synMAAPs produced a modest increase in capsid levels. However, synMAAP2, synMAAP4, synMAAP7, and synMAAP11 showed robust increases in secreted AAV particle numbers, 70% higher than wild-type MAAP. We then evaluated AAV particle heterogeneity using the SamuxMP mass photometer (Refeyn). While synMAAP variants increased the capsid-associated signal in the secretion assay as measured by ELISA (crude supernatant), this increase was not accompanied by a significant change in the affinity-purified particle abundance or mass distribution as analyzed by mass photometry (Fig. 3E). This supports the idea that the MAAP variants may affect the capsid-associated material present in the crude supernatant more than they affect the amount of purified intact AAV particles recovered by affinity chromatography. To ensure there were no differences related to packaged genomes, we evaluated the transduction of the virus resulting from synMAAP trans-complementation. Transduction assays using a luciferase reporter gene showed no functional differences among the variants (Fig. S2B). Based on these results, we prioritized synMAAP7 and synMAAP11 for further characterization.

We next asked whether these variants function across AAV serotypes. Previous work from our group demonstrated that MAAP8 trans-complementation restores secretion in a serotype-independent manner (11). Testing synMAAP7 and synMAAP11 with AAV1, AAV2, and AAV8, we observed a significant increase in the percentage of secreted vector for both candidates. SynMAAP11 consistently emerged as the lead variant, approximately 2.2-fold increase in the percentage of secreted vector genomes compared to wild-type MAAP across all tested serotypes, including AAV2, which is typically cell-pellet-associated (16) (Fig. 3F). Having established that synMAAP variants derived from the C-terminal domain library were best suited to enhance AAV secretion, we next sought to investigate their structural and mechanistic features.

### Biophysical analysis reveals C-terminal domain-based synMAAPs exhibit enhanced helical propensity and amphipathicity

To gain further insight into the biophysical properties of the enriched variants, we next turned to computational analyses. Previous predictions indicated that the MAAP C-terminal helix is amphipathic, having both polar and hydrophobic residues segregated on opposite faces of the helix. This motif is commonly associated with membrane binding, curvature induction, and protein trafficking, and has been implicated in the egress of other viruses (27–30).

**Fig 4:**
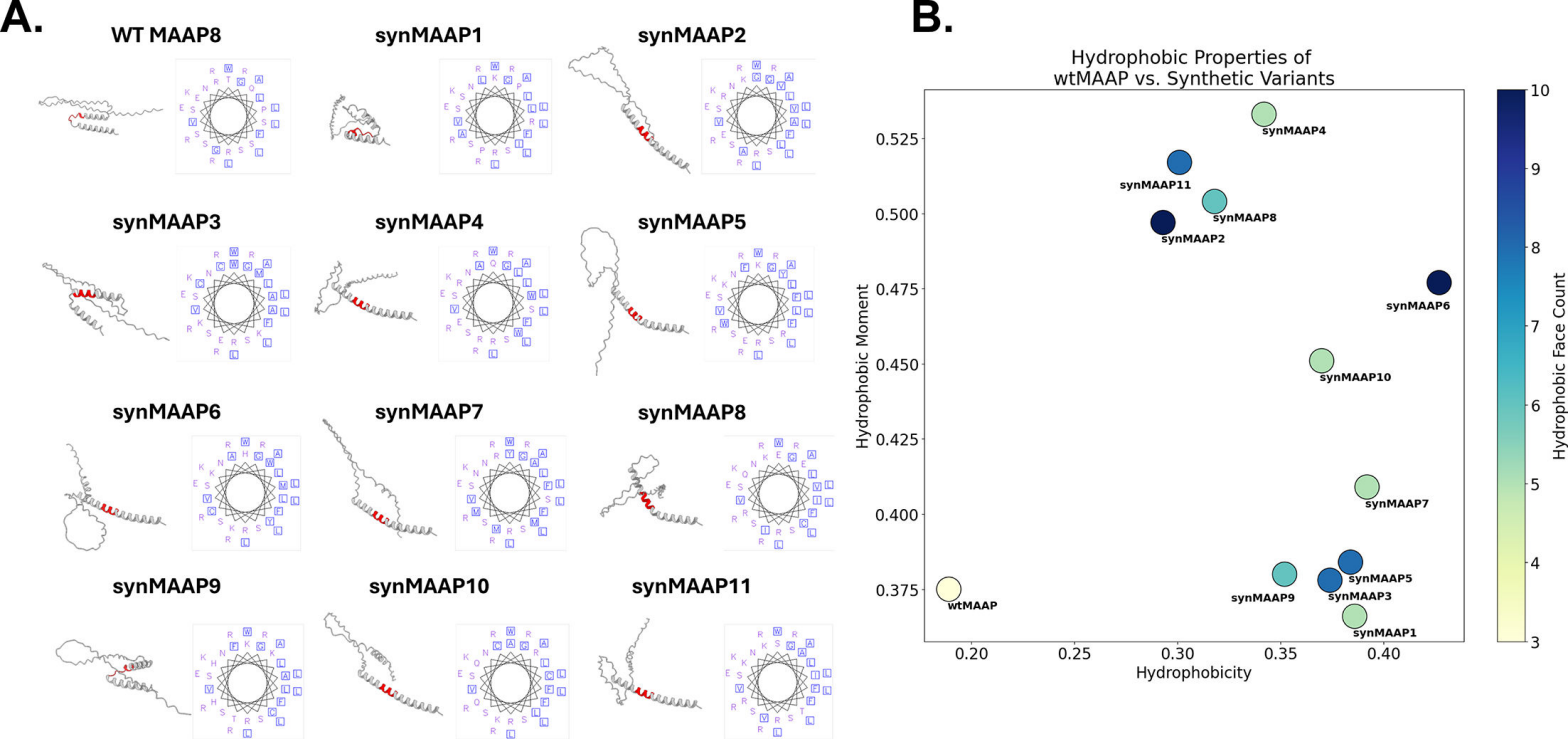
Evolved candidates reveal structural convergence with enhanced biophysical properties. (**A**) Structural predictions of engineered MAAP variants show additional structure surrounding the engineered region. Models were generated using AlphaFold3 and demonstrate structural convergence. The randomized library region is highlighted in red. Helical wheel projections confirm each variant contains an amphipathic helix, with hydrophobic residues highlighted in blue. (**B**) Biophysical properties were assessed to compare helix hydrophobicity and hydrophobic moment. The X-axis shows hydrophobicity, the Y-axis shows hydrophobic moment, and the colors indicate the number of residues within the hydrophobic face, as determined by HeliQuest.

We applied three analyses to characterize synMAAPs. First, AlphaFold3 structural predictions reveal that all but three variants (synMAAP1, synMAAP3, and synMAAP9) contained elongated α-helix relative to the wild-type protein. Next, we used pepwheel and HeliQuest prediction software to generate helical wheels, define the hydrophobic face from each helix, and quantify amphipathic and hydrophobic properties of the synMAAPs (Fig. 4A) (31, 32). Wild-type MAAP was predicted to have three amino acids within its hydrophobic face, whereas synMAAPs varied in range from five to ten. Consistent with this finding, the hydrophobic moment, a measure of amphipathicity, showed that wild-type MAAP scored 0.375, while all variants except synMAAP1 exhibited higher values, ranging from 0.378 to 0.533. Notably, some of the best-performing candidates (synMAAP2, synMAAP4, and synMAAP11) also had the highest hydrophobic moments. Finally, mean hydrophobicity scores were increased for all synMAAPs compared to wild-type MAAP, although no clear trend emerged (Fig. 4B).

Taken together, these results demonstrate that synMAAP variants broadly exhibit enhanced amphipathicity and hydrophobic profiles, and display increased helicity relative to wild-type MAAP, properties consistent with altered membrane-binding potential. Based on these findings, we next investigated whether engineered synMAAPs differ in their intracellular localization and trafficking.

### SynMAAPs display strikingly distinct intracellular trafficking and localization from wild-type MAAP

Having established that synMAAP variants enhance AAV secretion and display increased amphipathic and hydrophobic properties, we next asked whether these biochemical changes appear as differences in intracellular localization. MAAP has previously been suggested to associate with membranes and trafficking machinery, and while some interactors have been identified, the precise mechanism remains unclear (11, 12, 18). To address this, we examined the subcellular distribution of wild-type and synMAAP variants using confocal microscopy.

**Fig 5:**
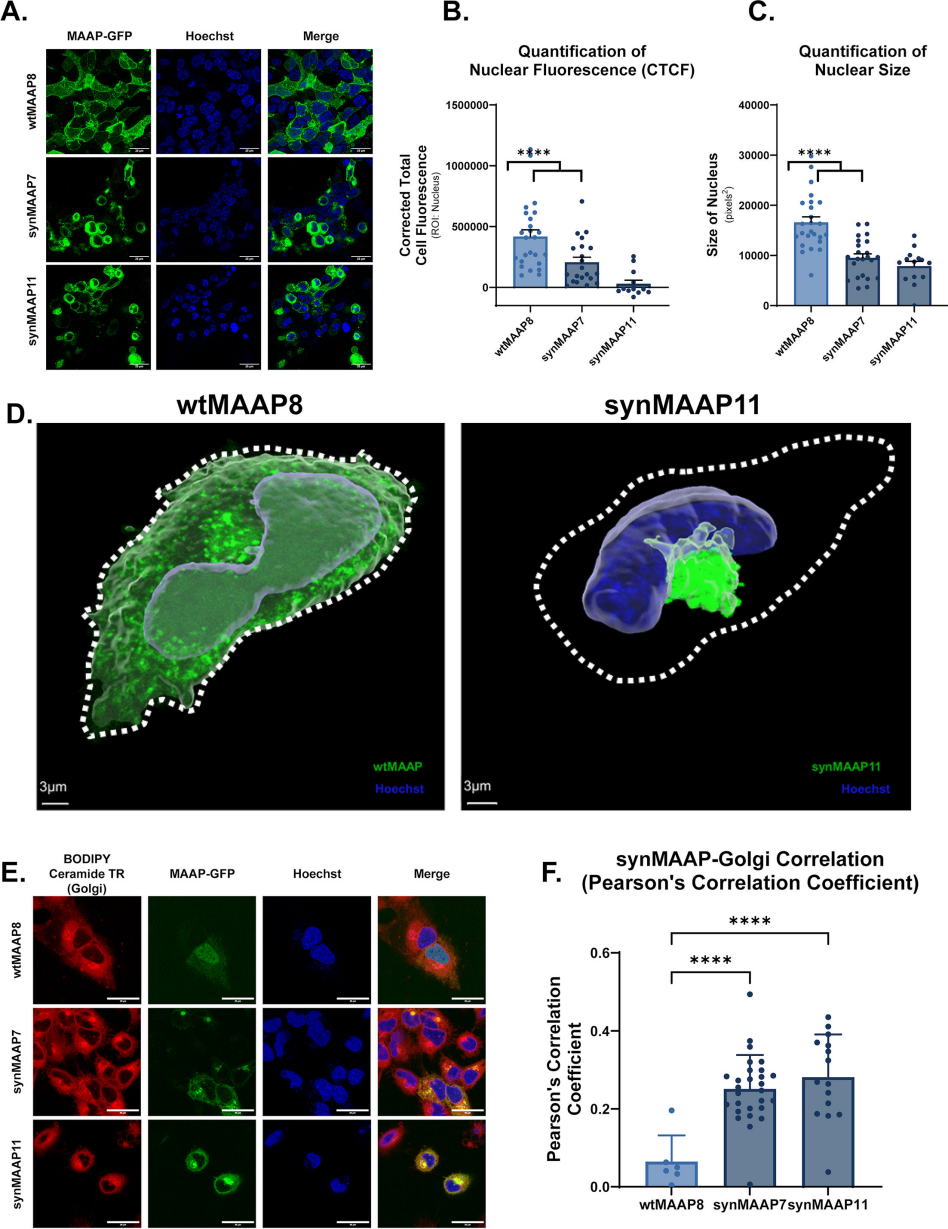
Live cell imaging reveals distinct localization differences between wild type and synMAAP. (**A**) Live-cell confocal imaging of HEK293 cells 24 hours post-transfection reveals distinct localization patterns of wild-type and synMAAP proteins. Wild-type MAAP displays a diffuse cytoplasmic and nuclear distribution, while synMAAP variants exhibit a concentrated signal in the perinuclear region. Scale bars = 25 µm (**B**) Nuclear fluorescence was quantified using a modified CTCF method. Wild-type MAAP shows a statistically significant increase in nuclear signal compared to synMAAP variants (*n* = 26 for WT, *n* = 23 for synMAAP7, and *n* = 14 for synMAAP11 cells per condition, mean ± SEM; significance was determined using one-way ANOVA with Dunnett’s post-test). (**C**) Nuclear size was determined by DAPI staining and found to be significantly reduced in synMAAP- expressing cells relative to those expressing wild-type MAAP (*n* = 26 for WT, *n* = 23 for synMAAP7, and *n* = 14 for synMAAP11 cells per condition, mean ± SEM; significance was determined using one-way ANOVA with Dunnett’s post-test). (**D**) Three-dimensional surface maps generated in Imaris illustrate the spatial distribution of wild-type and synMAAP fusion proteins. Scale bar = 3 µm. (**E**) To further evaluate localization patterns, live cell imaging of HeLa cells was performed using the same N-terminal fusion constructs. The Golgi apparatus was visualized with BODIPY-Ceramide TR, and nuclei were stained with Hoechst. Stronger co-occurrence was observed between the red (Golgi) and green (GFP) for the synMAAP transfection conditions. (**F**) To quantify this, Pearson’s correlation coefficient was calculated in ImageJ using the Just Another Colocalization Plugin (JACoP). Scale bar = 25 µm (*n* = 7 for WT, *n* = 28 for synMAAP7, and *n* = 15 for synMAAP11 cells per condition, mean ± SEM; significance was determined using one-way ANOVA with Dunnett’s post-test). ****P ≤ 0.0001.

To visualize localization, we generated fluorescent reporter constructs in which wild-type MAAP or synMAAP variants were fused to GFP or mCherry at the N-terminus. These constructs were validated for proper expression and function (by secretion assays) in HEK cells before use in microscopy (Fig. S3A and B). Confocal imaging revealed striking differences between wild-type MAAP and synMAAP variants (Fig. 5A). Wild-type MAAP displayed diffuse signal across the cytoplasm and nucleus, whereas all synMAAP variants showed strong perinuclear accumulation and complete absence of nuclear signal. Quantification of nuclear fluorescence by CTCF to determine signal abundance confirmed a statistically significant reduction in nuclear signal for synMAAP7 and synMAAP11 (Fig. 5B) and for all other variants evaluated (Fig. S3C and D). Notably, synMAAP11 yielded negative CTCF values, indicating nuclear fluorescence below background and consistent with near-complete exclusion from the nucleus. Because MAAP is membrane-associated, single-plane images from Z-stacks were analyzed to avoid artifacts from max-projection modeling (Fig. S3E and F). Representative videos further illustrate the localization patterns (Video S1).

Another consistent observation was a reduction in nuclear size across all synMAAP variants compared to wild type (Fig. 5C). This phenotype was also observed for all other synMAAP variants (Fig. S3G), and while increased cell death or reduced viability was not observed, suggesting nuclear size is not a consequence of toxicity, this phenotype remains unexplored. Three-dimensional models of cells were generated using surface mapping of Z-stacks of cells transfected with either synMAAP11 or wild-type MAAP. As seen in the single-plane images, MAAP shows diffuse localization in the wild-type conditions, while synMAAP is concentrated almost entirely in the perinuclear space (Fig. 5D).

We next sought to better understand the perinuclear localization of synMAAP proteins. Live cell imaging of HeLa cells stained with BODIPY Texas Red Ceramide, a live cell dye used to label the Golgi, and transfected with fluorescent MAAPs revealed a strong co-occurrence of synMAAP variants with the Golgi staining, whereas wild-type MAAP again displayed diffuse cytoplasmic and nuclear signal (Fig. 5E). Quantification by Pearson’s correlation analysis to measure co-occurrence of MAAP and Golgi showed significantly higher correlation coefficients for synMAAP variants relative to wild-type MAAP, consistent with moderate, but specific localization to the Golgi (Fig. 5F).

Collectively, this indicates that the synMAAP variants are excluded from the nucleus and preferentially accumulate at the Golgi, in contrast to the diffuse localization of wild-type MAAP. These differences can be attributed to the increased amphipathicity of the engineered variants and indicate that the synMAAPs are more efficient in engaging with secretory proteins, providing a potential mechanistic explanation for the enhanced vector egress.

### Proximity ligation analysis reveals the intracellular interactome of MAAP8

**Fig 6:**
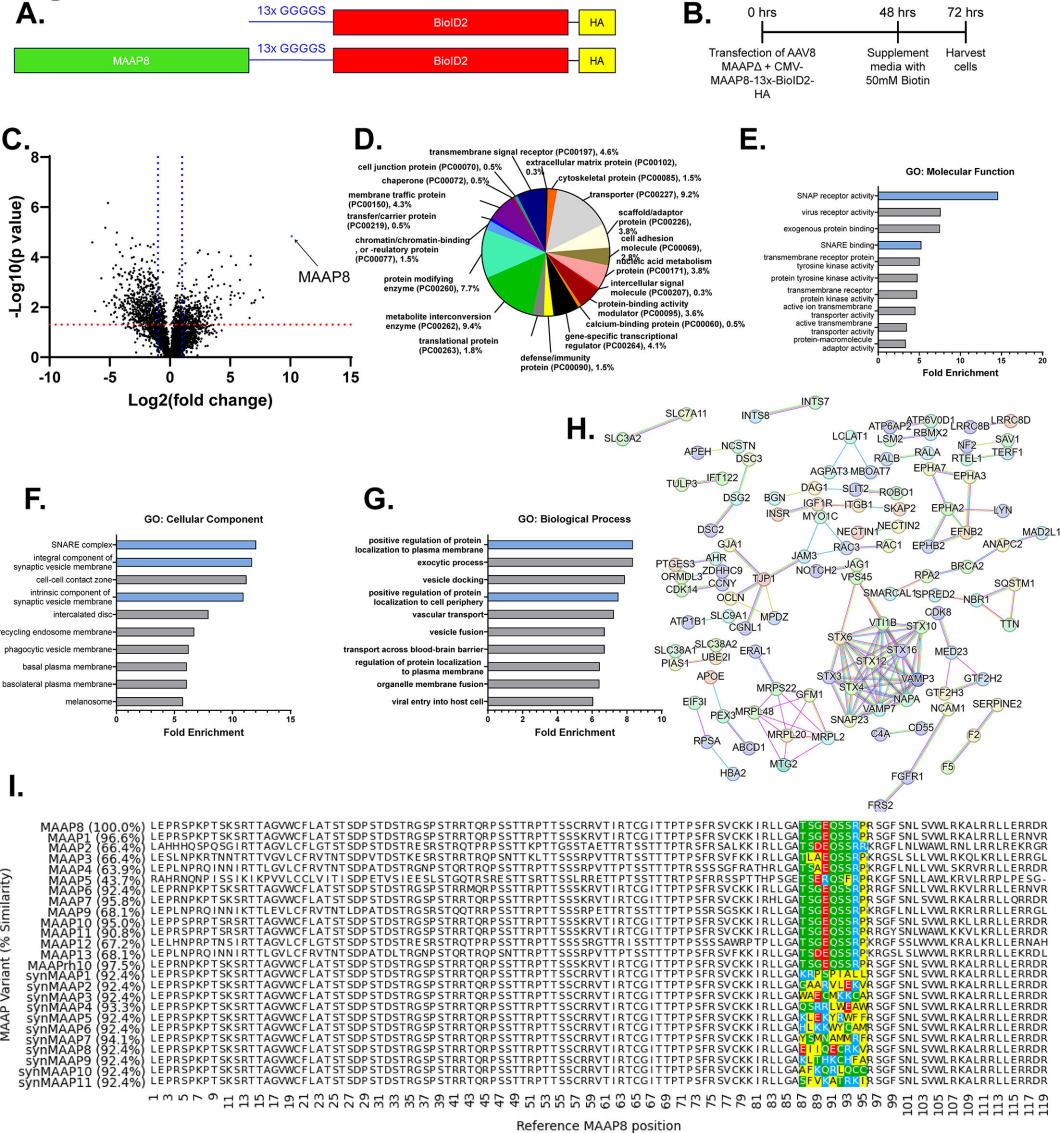
Proximity ligation assay reveals pathways involved in MAAP-mediated viral egress. (**A**) Schematic of 13×-BioID2 and MAAP8-13×-BioID2 fusion proteins. (**B**) Experimental timeline of the proximity ligation experiment. HEK293 cells were transfected with AAV8ΔMAAP, pHelper, and CMV-MAAP8-13×-BioID2 plasmids. Biotin (50 mM) was supplemented 48 hours post-transfection, and cells were harvested, and biotinylated proteins were purified using streptavidin-conjugated beads. (**C**) Volcano plot of proteins enriched in MAAP8-BioID2 samples. Y-axis represents the –log_10_(*P*-value), X-axis represents the log_2_(fold change[MAAP8-13×-BioID2/13×-BioID2]). (**D**) PANTHER protein class analysis of proteins enriched in the MAAP8 interactome relative to the BioID only control. (**E and F**) Gene ontology analysis for molecular function (**E**), cellular component (**F**), and biological process (**G**). (**H**) STRING network analysis showing clustering of SNARE-associated proteins within the MAAP8 interactome. (**I**) Protein sequence alignment of MAAP from common AAV serotypes and engineered variants.

To investigate the cellular pathways underlying MAAP8-mediated secretion, we performed BioID proximity labeling to identify host factors in close proximity to MAAP8. For this assay, MAAP8 was fused to biotin ligase BioID2 through a 13× GGGS linker, enabling the biotinylation of proteins within 25 nm (Fig. 6A) (33). MAAP8-BioID2 was expressed during recombinant AAV production to capture interactions in the context of viral assembly and secretion (Fig. 6B). Proteomic analysis of biotinylated proteins revealed nearly 400 candidates enriched in MAAP8-expressing cells compared to the no-MAAP control (fold change > 1.5; Fig. 6C). Functional classification using PANTHER showed abundant representation of metabolite interconversion enzymes (9.4%), transporters (9.2%), and protein-modifying enzymes (7.7%) (Fig. 6D). Gene ontology analysis for molecular function identified strong enrichment for SNAP receptor activity (~15-fold), which is consistent with the role of MAAP in viral secretion (Fig. 6E). Furthermore, cellular component GO analysis revealed enrichment in SNARE complex, synaptic vesicle membrane components, and cell-cell contact zones, each with >10-fold enrichment (Fig. 6F). Biological processes from the GO analysis were enriched for protein localization to the plasma membrane, exocytosis, and vesicle docking, each with ~8-fold enrichment (Fig. 6G). Taken together, these results suggest that MAAP8 engages with the host secretory machinery to facilitate viral egress, in line with our previous work establishing MAAP as an essential egress factor. Further investigation of the proteomic data set using STRING analysis revealed extensive enrichment of SNARE complex proteins, including NAPA, SNAP23, Syntaxin3, Syntaxin4, Syntaxin6, Syntaxin10, Syntaxin12, Syntaxin16, VAMP3, VAMP7, VPS45, and VTI1B (Fig. 6H).

Consistent with our hypothesis that sequence and structural diversity of naturally derived MAAPs will likely lead to distinct intracellular interactomes, the observed list for MAAP8 is strikingly different from those previously identified for MAAP2 (from AAV serotype 2). Previous studies have demonstrated the differences in secretion kinetics across serotypes (15, 16). Given the established role of MAAP as a viral egress factor, this phenotype is almost certainly due to differences in the MAAP protein among serotypes. In fact, sequence alignment of common MAAP proteins (1–13, rh10) to MAAP8 reveals similarities ranging from 44% similar for MAAP5 to 97% similar to MAAP1 and MAAPrh10 (Fig. 6I). This level of sequence divergence is on par with what is observed in capsid evolution, where changes to the viral surface result in serotype-specific engagement of different entry receptors. By the same logic, different MAAPs are likely to interact with different components of the host trafficking machinery and hijack separate egress mechanisms to complete the viral lifecycle. Some MAAPs may have a bias toward canonical secretion pathways, while others may leverage or develop new pathways for viral egress.

Our results demonstrate that MAAP associates (directly or indirectly) with intracellular vesicular trafficking-associated proteins, including members of the SNARE complex, implying a role for these host factors in viral MAAP-mediated secretion. However, because proximity ligation alone cannot establish the function of these proteins in the context of enhanced secretion, we asked whether synMAAPs have similar dependencies and whether SNARE members are required to support viral egress.

### Functional gene deletion highlights key differences in synMAAP11 vs MAAP8 dependence on SNARE proteins for AAV egress

**Fig 7:**
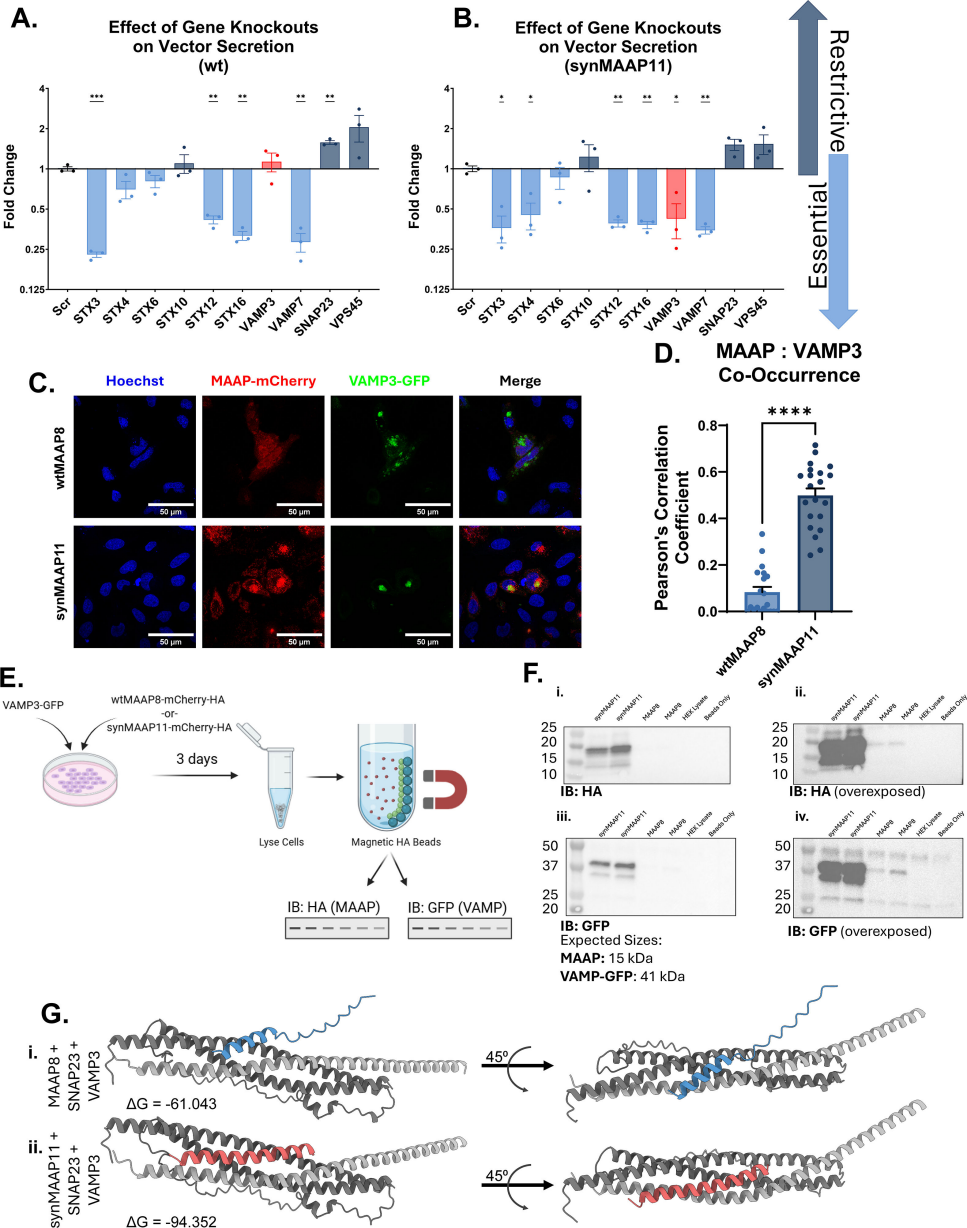
Functional testing of SNARE proteins in MAAP-mediated secretion and characterization of VAMP3-MAAP interactions. (**A and B**) rAAV secretion in CRISPR knockout lines targeting SNARE proteins identified by BioID. Titers were measured in the presence of MAAP8 or synMAAP11 and normalized to scramble controls. FC > 1 indicates the factor is restrictive for secretion, while FC < 1 indicates it is essential for secretion. (**C**) Confocal images illustrating VAMP3 distribution in cells expressing MAAP8 or synMAAP11. Scale bar = 50 µm. (**D**) Pearson’s correlation coefficient analysis of VAMP3 co-occurrence with MAAP8 or synMAAP11 (*n* = 20 for WT and *n* = 21 for synMAAP11 cells per condition, mean ± SEM; significance was determined using an unpaired t-test). ****P ≤ 0.0001. (**E**) Coimmunoprecipitation workflow for evaluating VAMP3 interactions with MAAP. (**F**) Western blots showing increased VAMP3 coprecipitation with synMAAP11 relative to MAAP8. Given the intensity of the bands for synMAAP11 (panels i and iii), images were overexposed to capture signal from the MAAP8 conditions (panels ii and iv). (**G**) AlphaFold3 structural predictions refined by Rosetta FastRelax of the predicted SNAP23/VAMP3/MAAP8 (i) or SNAP23/VAMP3/synMAAP11 complex (ii). Calculated ΔG values are listed below the respective models and indicate more favorable binding for synMAAP11. MAAP8 (blue), synMAAP11 (red), SNAP23 (dark gray), and VAMP3 (light gray).

To functionally evaluate the role of SNARE proteins in MAAP-mediated secretion, we generated knockout cell lines for selected BioID candidates. Using CRISPR/Cas9, we targeted SNAP23, VAMP3, and multiple syntaxins, and then quantified recombinant AAV secretion in the presence of either MAAP8 or synMAAP11, normalized to scramble controls (Fig. 7A and B). Overall, syntaxin knockouts (KO) produced comparable effects across both groups. Consistent with previously published results, SNAP23 KO enhanced the secretion previously reported by the Qiu group (18).

Several target gene candidates showed little to no difference upon KO in secretion between wildtype and synMAAP11. Interestingly, STX10, STX12, STX16, and VAMP7 KO all showed similar decreases in fold change in secretion across conditions. STX3 and STX4 KO each showed decreases in secretion, but the effect was stronger for opposite conditions. Specifically, STX3 KO reduced secretion more effectively in the case of MAAP8, whereas STX4 KO reduced fold change more for synMAAP11. As described earlier, SNAP23 KO appears to have a restrictive effect, with increased secretion for both MAAP8 and synMAAP. The most notable result is VAMP3 KO, wherein MAAP8-mediated secretion was unchanged, but synMAAP11-mediated secretion showed a significant reduction. Given the established role of VAMP3 in receptor recycling, secretory vesicle fusion, and retrograde transport to the Golgi, we next investigated its subcellular distribution in this context.

Confocal microscopy revealed distinct patterns of VAMP3 localization for synMAAP11 and wild-type protein (Fig. 7C). With MAAP8, VAMP3 had a diffuse vesicular distribution. However, co-expression with synMAAP11 revealed enrichment in foci, brighter intensity, and loss of the vesicular morphology. Pearson’s correlation analysis confirmed greater co-occurrence between VAMP3 and synMAAP11 compared to MAAP8, indicating a moderate yet specific co-occurrence between VAMP3 and synMAAP11 (Fig. 7D). Given the enhanced localization with synMAAP11 and VAMP3 observed by microscopy, we next asked whether these proteins are physically associated.

### Biochemical analysis and computational modeling provide insights into a plausible mechanistic model based on synMAAP11-SNARE interactions

To evaluate interactions between MAAP and VAMP3, we performed co-immunoprecipitation experiments. Briefly, GFP-tagged VAMP3 was co-transfected with either mCherry-tagged wtMAAP8-HA or mCherry-tagged synMAAP11-HA, and cell lysate was subjected to HA affinity purification (Fig. 7E). Compared to wtMAAP8, synMAAP11 showed a remarkable increase in precipitation with VAMP3, with both replicates displaying increased HA and GFP signal (Fig. 7Fi and iii). Upon overexposure of the blot, weak but detectable bands for wtMAAP8 were observed, consistent with the reduced signal noted in confocal microscopy (Fig. 7Fii and iv). These results indicate that synMAAP11 may enhance the interaction with VAMP3. To orthogonally validate this, we turned to computational modeling.

Previously published data show that VAMP3 interacts with syntaxin 1, syntaxin 4, SNAP23, and SNAP25 (34–36). Other groups have recently shown that VAMP3 forms ternary complexes with syntaxin 4 and SNAP23 (37). Given that both syntaxin 4 and SNAP23 appeared in our BioID hits, we sought to evaluate the predicted binding of synMAAP11 in this complex. AlphaFold3 provides predictions of protein structures, but the side-chain conformations (rotamers) and protein backbone are not always in their preferred orientation (38). To address this, we used Rosetta FastRelax to refine the AlphaFold3 prediction and minimize steric clashes (39). Then, we calculated the change in Gibb’s free energy (ΔG) of the interaction between the helical region of MAAP or synMAAP11 and the host protein complex. AlphaFold3 was unable to reliably capture the structure of the four-protein complex comprising syntaxin 4, SNAP23, VAMP3, and (syn)MAAP, resulting in large deviations in the predicted backbone conformations. For this reason, we proceeded with binding predictions based on SNAP23, VAMP3, and MAAP proteins without syntaxin 4 (Fig. 7G).

A negative ΔG implies more favorable binding energy, which could suggest stronger or more stable protein-protein interactions. SynMAAP11 has a predicted ΔG of −94.352 compared to a ΔG of −61.043 for wild-type MAAP (Fig. 7G). Interpretation of this finding with the co-immunoprecipitation results is consistent with, but does not independently establish, greater stability for the VAMP3/SNAP23/STX4 complex compared to wtMAAP interactions, potentially explaining its improved secretion and altered localization. The similarity of synMAAP proteins to cellular SNARE proteins may facilitate interactions with the SNARE complex and result in enhanced egress of viral particles.

## DISCUSSION

In this study, we demonstrate that structure-guided evolution of the AAV MAAP yields synthetic variants that significantly enhance vector secretion in a serotype-independent manner. Using targeted mutagenesis libraries of MAAP8 and iterative selection through two different tracks, transfection and serial viral passaging, we identified thousands of enriched sequences. Top-performing candidates were selected using structural predictions and biophysical considerations. Individual evaluation revealed that multiple synMAAP variants increased the proportion of secreted vector genomes relative to wild-type MAAP. Improvements were also observed across multiple serotypes. To further understand structure-function determinants, we focused on the most potent candidates—synMAAPs with extended C-terminal amphipathic helices, displaying increased helical propensity and hydrophobic moment. These synMAAPs demonstrated a drastic shift in intracellular localization, from diffuse global localization toward highly selective sequestration within the Golgi secretory compartment. Interactome mapping of synMAAPs through proteomic analysis suggests altered and intensified host trafficking machinery engagement by synMAAPs. Specifically, it appears that the synMAAPs engaged VAMP3, a critical member of the SNARE complex.

From a structural and basic AAV biology standpoint, our findings emphasize the critical role of the MAAP C-terminal domain in mediating viral egress. Previous studies from our lab have shown that the MAAP C-terminus is required in part for protein stability (11). Further dissection of this region demonstrated that two distinct segments within the C-terminus have different consequences when deleted. Removing the residues upstream of the C-terminal helix had no significant impact on viral production or egress, while deleting the core of the helix was deleterious to both protein production and viral egress (11). The current study validates these past findings, but more importantly, underscores the role of the helical and amphipathic nature of the C-terminus in mediating AAV egress. Our results also provide a structural explanation for a previously reported, engineered MAAP2 variant lacking a C-terminal helix that appears to support an increase in total viral genomes, but with minimal impact on secreted vector (40).

From a biochemical perspective, proximity ligation analysis revealed that one of our variants, synMAAP11, appears to engage key components of the SNARE complex. Intriguingly, structural prediction of wtMAAP8 demonstrates a helix-turn-helix in the C-terminal region, while several variants demonstrate an elongated C-terminal helix. These closely resemble many SNARE proteins, such as VAMP3, and may also enhance the interaction with other SNARE proteins. More specifically, SNARE proteins are essential factors for intracellular membrane fusion and vesicle trafficking (41). Individually, they are composed of a variable N-terminal region that dictates properties of the specific protein, one or more coiled-coil SNARE motifs consisting of heptad repeats forming amphipathic α-helices, and an optional C-terminal transmembrane domain (42). Specifically, these features bear a striking similarity to the structure of MAAP, which contains a disordered N-terminus, T/S-rich linker region, and a C-terminal amphipathic α-helix. Thus, it is possible that the helical extension of synMAAP variants functions as a SNARE mimetic and potentiates the “zippering” activity of SNARE complex proteins known to drive vesicular fusion. Such a model is supported by many viruses, which have evolved to mimic or co-opt SNARE machinery for completion of their lifecycle. For example, certain viruses contain fusion proteins forming coiled-coil domains like the HA protein of influenza, the gp41 protein of HIV, and the GP2 protein of Ebola (43). Other viruses, including herpesviruses and poxviruses, interact directly with the host SNARE machinery to reroute vesicle trafficking toward secretory pathways (44).

From the viewpoint of intracellular trafficking, our data show that synMAAP variants appear to enhance AAV secretion by rewiring host vesicle trafficking through strengthened interactions with VAMP3. SynMAAP11 shows a striking pattern of accumulation within the Golgi, hijacking the secretory pathway by directly engaging VAMP3. Earlier studies with MAAP2 suggest that deletion of SNARE family members such as SNAP23 or STX7 increases viral titers through a different mechanism by preventing lysosomal trafficking of the late endosome, which, in turn, prevents rAAV vector degradation (18). Moreover, no direct engagement of the SNARE machinery was demonstrated. Our findings suggest that robust viral egress follows a distinct mechanism, wherein synMAAPs can function as direct binding partners of SNARE proteins and hijack vesicle fusion pathways leading to extracellular secretion/export.

While these studies have focused specifically on AAV9 and other clinically relevant serotypes, understanding the role of MAAP more broadly could help elucidate viral egress of other members of the *Parvoviridae* family. Importantly, MAAP has been identified as an alternative ORF within members of the *Dependoparvovirus* genus, specifically *dependoparovirus A, and in AAV5, a strain of dependoparvovirus B,* along with porcine AAVs. Previous studies and our results show that MAAP (and synMAAPs) function broadly across AAV serotypes. Combined with the host factors, we and others have identified that the enhanced secretion phenotype is expected to extend across dependoparvoviruses. However, MAAP is not present in all parvovirus genera, and its role outside this group remains unclear.

Taken together, we propose a model where the extended amphipathic C-terminal helix of synMAAPs may act as an additional SNARE-like motif that associates with VAMP3-positive secretory vesicles at the Golgi. By engaging VAMP3, synMAAP11 may help stabilize the SNARE complex and/or potentiate membrane fusion events involved in cellular secretion, with AAV particles co-opting this secretory “highway” for rapid egress. While additional investigation of cellular secretion pathways is needed to dissect such mechanism(s), our model provides a general framework for understanding how synMAAPs may reprogram host trafficking machinery for viral egress. It is noteworthy to mention that similar patterns have been reported for human cytomegalovirus (HCMV), where VAMP3 is essential for virion maturation and egress through the Golgi-derived viral assembly compartment (vAC) (45). In HCMV, VAMP3 knockout impairs trafficking without affecting viral replication or protein synthesis. The perinuclear localization of synMAAP11, co-occurrence with Golgi markers, and potential interactions (directly or indirectly) with SNARE complex members such as VAMP3 underscore similarities between such egress mechanisms.

From an application viewpoint, improving the efficiency of recombinant AAV vector production remains a challenge for gene therapies. The synMAAPs developed in the current study could potentially improve process development at an upstream level, improving the yield of full genome packaging particles secreted into the media as well as the downstream level, potentially decreasing the burden on purification steps without the need to process producer cell lysate and contaminants. Specifically, integrating regulated synMAAP expression into helper plasmids or stable cell lines and incorporating it within manufacturing workflows could increase the proportion of functional, genome-containing rAAV particles. Furthermore, the extraction and purification of AAV particles secreted into the media could enable optimization of continuous perfusion manufacturing processes with the overall goal of improving efficiency and decreasing cost. Such engineered cellular egress factors that potentiate the secretory efficiency may also be applicable for the manufacturing of other recombinant biologics.

Several limitations of the study should be noted. The overall basis for this work hinges on overexpression and trans-complementation of synMAAPs. It is plausible that such an approach may not fully recapitulate biologically relevant MAAP mechanisms employed during the natural infectious AAV lifecycle. Relatedly, the cell biology of MAAPs and synMAAPs outlined in the current study warrants significant additional investigation to determine a more complete picture of viral egress. From a manufacturing complexity standpoint, integration of synMAAPs into already complex multi-component process development workflows will require further optimization. Specifically, the kinetics and dynamics of enhanced cellular secretion may vary significantly and require further regulation on a larger scale. It is also important to note that the membrane fusogenic activity of synMAAPs and the resulting increase in secretory output will impact the overall health of producer cell lines and require nutritional replenishment strategies.

## Data Availability

Data Set S1 and Data Set S2 include next-generation sequencing data for different synMAAPs selected through infectious cycling and mass spectrometry data from proximity labeling studies. Both data sets include raw read counts, fold changes, and *P* values compared to respective controls.

## SUPPLEMENTAL MATERIAL

Data Set S1 jvi.00026-26-s0001.xlsx NGS sequencing data.

Data Set S2 jvi.00026-26-s0002.xlsb MS proximity labeling data.

Supplemental figures jvi.00026-26-s0003.pdf Fig. S1 to S3.

Video S1 jvi.00026-26-s0004.mp4 Differential intracellular localization patterns of wild-type MAAP8 and SynMAAP11 with the Golgi compartment (Golgin97 marker).

ASM does not own the copyrights to Supplemental Material that may be linked to, or accessed through, an article. The authors have granted ASM a non-exclusive, world-wide license to publish the Supplemental Material files. Please contact the corresponding author directly for reuse.
