# Supplementary material for: Natural and evolved membrane-associated accessory proteins differentially engage SNARE machinery for AAV egress
Source: J Virol. 2026 Jun 10;100(7):e00026-26. doi: 10.1128/jvi.00026-26 (PMC13386907; doi:10.1128/jvi.00026-26)
Supplement: Supplemental figures — Fig. S1 to S3. [file jvi.00026-26-s0003.pdf]

Supplementary Figure 1

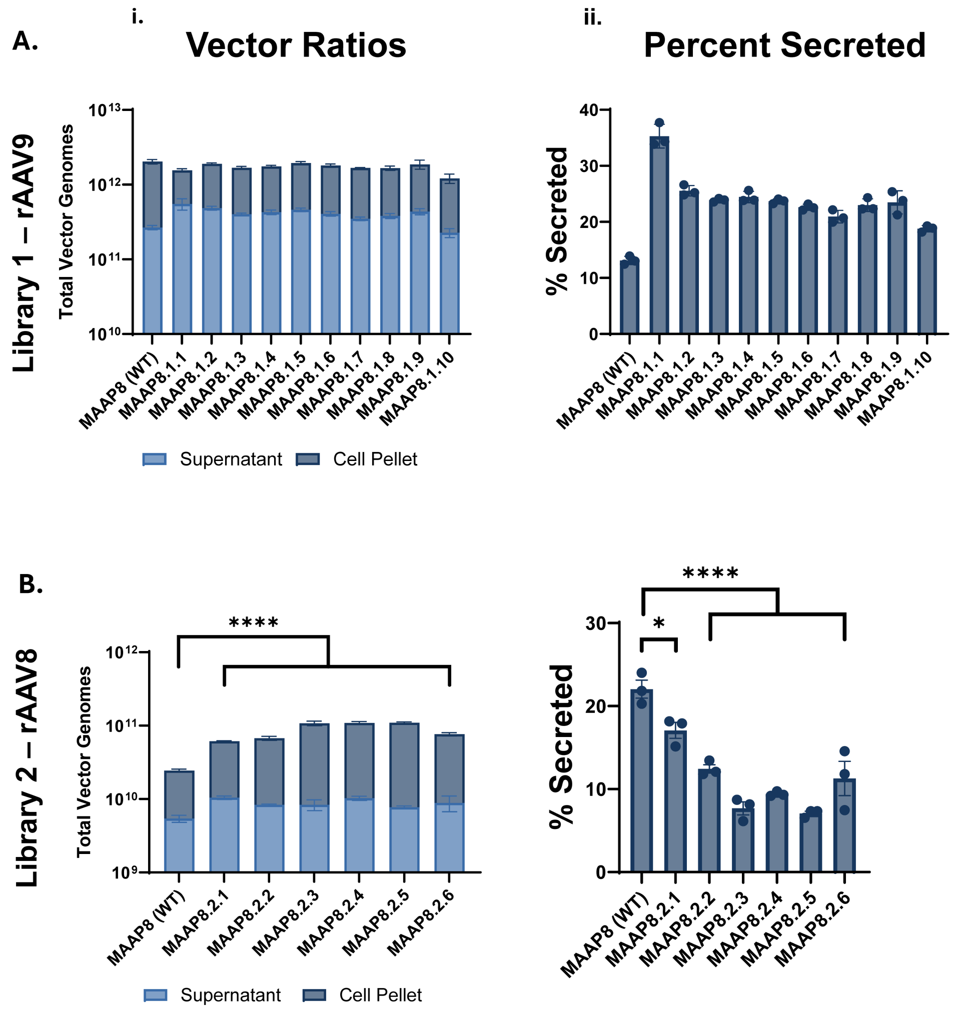

Supplementary Figure 1: Evaluation of candidates from remaining MAAP libraries. A (Library 1). (i) Quantitative PCR titers of virus of secreted and cell-retained vector genomes at three days post-transfection. Select variants show increased secretion at three-days post transfection. N=1. **B (Library 2).** (i) Quantitative PCR titers of virus of secreted and cell-retained vector genomes at three days post-transfection. Variants show increased genomes within the cell (statistics plotted) and decreased secretion (not statistically significant) at three-days post transfection. Two-way ANOVA with Dunnett's posttest for secreted and retained fractions, and one-way ANOVA with Dunnett's posttest for percent secreted. Data shown as mean ± SEM. Significance: * $p \leq 0.05$; **** $p \leq 0.0001$. N=3.

Supplementary Figure 2

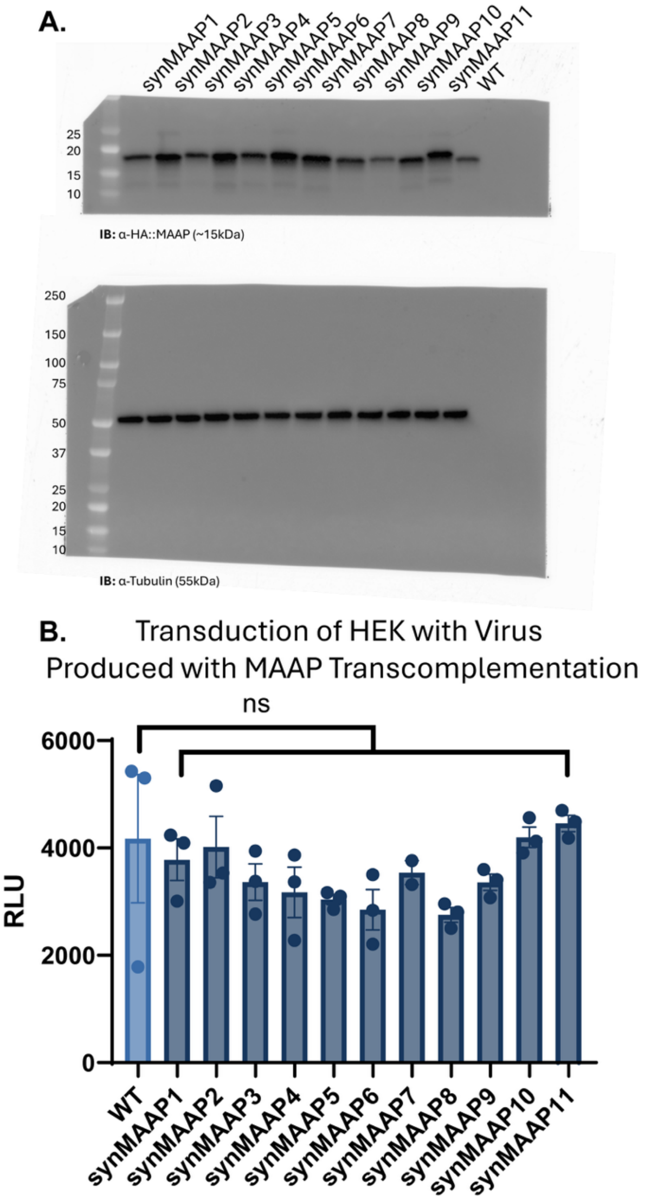

Supplementary Figure 2. Protein expression of MAAP variants and transduction activity of resulting purified virus.

A. Western blot analysis of variants expressed from the EF1α-driven cassette in HEK293 cells, confirming protein expression across all candidates selected for follow-up. **B.** Luciferase transduction assay demonstrating equivalent functional activity among AAV produced from MAAP transcomplementation. Significance was determined using one-way ANOVA, with Dunnett's posttest. Data are presented as mean values ± SEM

Supplementary Figure 3

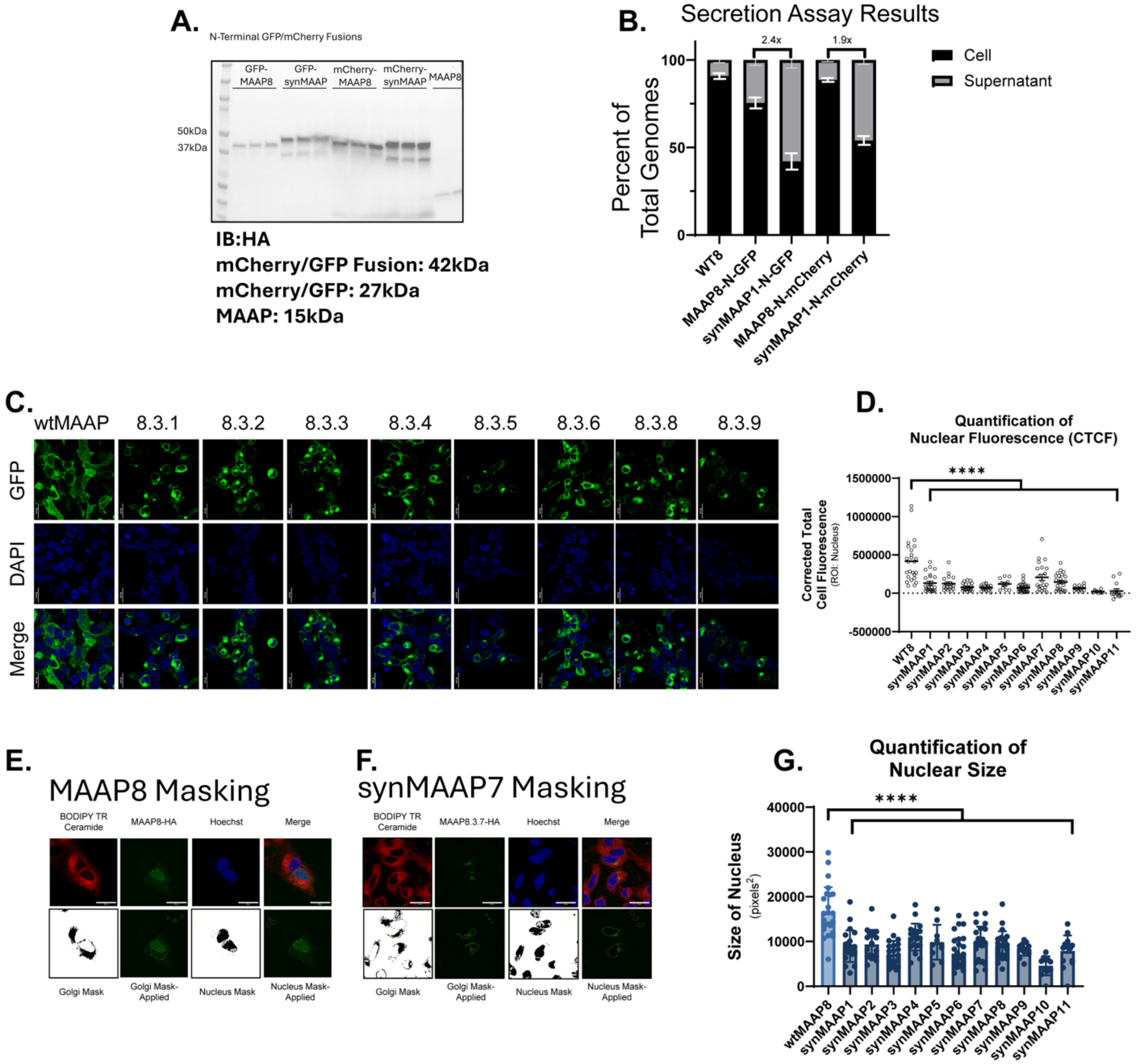

Supplementary Figure 3. Expression, secretion, and localization characteristics of fluorescent MAAP fusions.

A. Western blot confirming expression of GFP- or mCherry-tagged wildtype and synMAAP fusion constructs in HEK293 cells. **B.** Secretion assay demonstrating that N-terminal fluorescent tagging does not disrupt MAAP function, with all fusions retaining secretion activity. **C.** Representative confocal images from live HEK293 cells expressing wildtype or synMAAP fusion constructs, illustrating distinct intracellular localization patterns. Scale bar=25 µm. **D.** Quantification of nuclear fluorescence using corrected total cell fluorescence (CTCF), corresponding to the images in panel C. (n=25 for wtMAAP and n

ranges between 10 and 31 for remaining variants; mean ± SEM; significance was determined using one-way ANOVA with Dunnett's posttest). **E-F.** Masking parameters and representative single-plane slices from Z-stack acquisitions. **G.** Quantification of nuclear size for each variant, showing consistent reductions across synMAAP constructs. (n=25 for wtMAAP and n ranges between 10 and 31 for remaining variants; mean ± SEM; significance was determined using one-way ANOVA with Dunnett's posttest).
